# Supplementary material for: Family rejection of non-hetero sexuality–Sexual orientation and behavior anonymity among sexual minority men in slum communities-BSGH 001
Source: PLOS Glob Public Health. 2023 Dec 1;3(12):e0001659. doi: 10.1371/journal.pgph.0001659 (PMC10691700; doi:10.1371/journal.pgph.0001659)
Supplement: Attachment — Submitted filename: SMM.docx [file pgph.0001659.s001.docx]

Thank you for inviting me to review this manuscript, which I have read with great pleasure. Anonymity/ non-disclosure and disclosure of sexual identity make an important contribution to SMM literature and advocacy efforts. However, the manuscripts would do with some review to for coherency.

Some comments for consideration;

Methodology: explain how the phenomenological approach was used, and how it has been applied to the findings.

Some themes and related analysis is not clear:

Your first theme shows why they fear. But as you move on it is about the causes of fear of disclosing /challenges of disclosing. If this is the structure, make it clear in the introduction of the findings section>

Page 13/14: Theme: Fear of deviation from culture is communicating something that is not clear. Review the section for clarity.

Page 12/13. Theme: Fear of social media- using quotes used from conventional media (radio, television, and newspapers) makes me feel you should rename the theme to Fear of the media, this way gives you the opportunity to write about any media.

Page 11/12: Theme: Fear of physical assault from family’s perception of SMM activity. Work on coherence. You mix the fears and coping strategies: I suggest, first, write about the fears, and second the coping mechanisms.

Page 15/16: Theme: Criminalization of SMM activities by LGBTQ+ legislation. This section is well-written, and clear. However, provide some brief information about the bill.

**Page 16. The sentence: The country's legislative focus against LGBTQ+ through existing laws and bills did nothing to address the real challenges of the economy but facilitated the unfair treatment of SMM in the country**. Do the law and bills to promise to address the real challenges of the economy?

Page 16: The sentence: **The continuous push for legislation against LGBTQ+ was cited as why they chose not to disclose their gender identity and sexual orientation/behavior to their friends or families.** You make a good point but needs to be unpacked.

Discussion: Try not to introduce new literature or references in the discussion section. Instead, you can introduce the new literature in the introduction section.
