# Supplementary material for: Family rejection of non-hetero sexuality–Sexual orientation and behavior anonymity among sexual minority men in slum communities-BSGH 001
Source: PLOS Glob Public Health. 2023 Dec 1;3(12):e0001659. doi: 10.1371/journal.pgph.0001659 (PMC10691700; doi:10.1371/journal.pgph.0001659)
Supplement: Attachment — Submitted filename: Response to Reviewers.doc [file pgph.0001659.s002.doc]

07/08/2023

Dear Reviewers,

Re: Manuscript ID: [PGPH-D-23-00095] Title: "Family Rejection of Sexuality – Sexual and Gender Anonymity among Sexual Minority Men in Slum Communities"

I am writing to express my deepest gratitude for your thorough review of our manuscript titled "Family Rejection of Sexuality – Sexual and Gender Anonymity among Sexual Minority Men in Slum Communities." Your insightful comments and suggestions have immensely contributed to enhancing our study, and we appreciate the time and effort you dedicated to reviewing our work.

I am pleased to inform you that we have addressed all the concerns raised by Reviewer One, and I would like to outline the specific revisions made in response to their valuable feedback:

1. **Reviewer 1:** Your first theme shows why they fear. But as you move on it is about the causes of fear of disclosing /challenges of disclosing. If this is the structure, make it clear in the introduction of the findings section. **Response:** "Fear" in the first theme has been rewritten to indicate that the fear discussed is directed towards the "fear of disclosing" rather than a "general fear." This revision is appreciated as the intended focus and context are accurately conveyed.
2. **Reviewer 1:** Page 13/14: Theme: Fear of deviation from culture is communicating something that is not clear. Review the section for clarity.

**Response:** This section has been rewritten to communicate how SMM who expressed feminine mannerisms were criminalized and their behaviors not culturally shared by inhabitants in the slums.

3. **Reviewer 1:** Page 12/13. Theme: Fear of social media- using quotes used from conventional media (radio, television, and newspapers) makes me feel you should rename the theme to Fear of the media, this way gives you the opportunity to write about any media.

**Response:** The phrase "fear of creating an unconducive environment for SMM" has been modified to reflect the "fear of media," which is a more precise representation of the concerns expressed by the participants.

4. **Reviewer 1:** Page 11/12: Theme: Fear of physical assault from family's perception of SMM activity. Work on coherence. You mix the fears and coping strategies: I suggest, first, write about the fears, and second the coping mechanisms.

**Response:** The theme "fear of physical assault from family's perception of SMM activity" has been revised to highlight the fears experienced by sexual minority men first, followed by their coping mechanisms.

5. **Reviewer 1:** Page 15/16: Theme: Criminalization of SMM activities by LGBTQ+ legislation. This section is well-written, and clear. However, provide some brief information about the bill.

**Response**: In response to the reviewer's suggestion, we have included a brief description of the anti-gay bill in the introduction of the paper. This addition provides necessary background information and serves as a reference point for the subsequent discussion on the theme "Criminalization of SMM activities by LGBTQ+ legislation."

6. **Reviewer 1:** Page 16. The sentence: The country's legislative focus against LGBTQ+ through existing laws and bills did nothing to address the real challenges of the economy but facilitated the unfair treatment of SMM in the country. Do the law and bills to promise to address the real challenges of the economy?

**Response:** The sentence in question, "The country's legislative focus against LGBTQ+ through existing laws and bills did nothing to address the real challenges of the economy but facilitated the unfair treatment of SMM in the country," has been revised to specify that it refers to the criminal code under section 104 of the Ghanaian constitution and its implications on sexual orientation/behavior.

7. **Reviewer 1:** Page 16: The sentence: The continuous push for legislation against LGBTQ+ was cited as why they chose not to disclose their gender identity and sexual orientation/behavior to their friends or families. You make a good point but needs to be unpacked.

**Response:** This review on the push for LGBTQ+ legislation has been unpacked to show how this creates a situation for MSM not to disclose their sexual orientation/behavior in Ghanaian slums.

8. **Reviewer 1:** Try not to introduce new literature or references in the discussion section. Instead, you can introduce the new literature in the introduction section.

**Response:** We incorporated new literature in the discussion section to address emerging issues derived from the findings of our study. This addition strengthens the significance of our research and enriches the contextual understanding of sexual and gender anonymity among sexual minority men in slum communities.

Moving on to the concerns raised by Reviewer Two, I am delighted to inform you that we have adequately addressed each point, and the following revisions have been made:

1. **Reviewer 2:** The authors state they "aim to explore how impoverished communities and families contribute to SMM disclosure of their gender identity, orientation and sexual behaviors". However, the impact of poverty or living in slum communities on disclosure/nondisclosure of sexual orientation/behaviors is not explored, although the impact of family and religious affiliations on disclosure is discussed in detail. The authors can thus consider revising the aim.

**Response:** As recommended by the reviewer, we have revised the aim of the study to align it more precisely with the research aim to explore the impact of family on SMM disclosure of their sexual orientation/behavior in the selected slum communities.

2. **Reviewer 2:** Throughout the manuscript, the term' gender identity' appears, while the inclusion criteria state that all the participants "must self-identify as a cis-gender man". Probably the authors meant 'gender expression' (and not 'gender identity') in most contexts, even though, to add to the confusion, one participant reported, "I don't attend church because of my gender identity". The authors need to clarify how the term' gender identity' fits within the context of conducting research among cisgender-identified MSM.

**Response:** We acknowledge the reviewer's suggestion and have incorporated the term "gender expression" to reflect the indigenous identities of sexual minority men.

3. **Reviewer 2:** Some background information about the percentage of those who practice different religions can be added to the Introduction section as part of understanding the religious contexts.

**Response:** We have included the data on the religious composition of participants in the methodology section, specifically under the subsection titled "Participants."

4. **Reviewer 2:** Most of the participants refer to themselves as "MSM", although the authors do use the term "a member of the LGBTQ community" as well. While the term "MSM" itself can be an identity, I suspect that many of the participants may have some kind of indigenous identities based on their sexual role and gender expression. In fact, one participant has been quoted to be saying "I don't have feminine features and I also don't have feminine friends. And so, they don't know". Apparently, feminine gender expression is seen as a proxy for sexual
5. orientation/identity. Can the authors discuss about these aspects (indigenous identities, role of gender expression in labelling someone as MSM)?

**Response:** The term "feminine" was mentioned by sexual minority men to illustrate how indigenous cultural and gender norms in Ghana, particularly within the slums, stigmatize men with feminine features or men associating with feminine men as MSM. We have clarified that the term "feminine" to reflect the expressions associated with

femininity and the potential compromises to an MSM's anonymity or safety when associating with feminine men or engaging in feminine behaviors. This clarification ensures that the intended meaning is accurately conveyed. Feminine gender expressions within the context of this study, and the general notion in the sampled slums and among participants were proxies to non-cisgender and non-heterosexual orientation/behaviors.

6. **Reviewer:**  I could not locate any theoretical frameworks (e.g., minority stress theory) being mentioned by the authors even in the Discussion section. Although not mandatory, the authors can consider referring to appropriate theoretical frameworks to strengthen the contribution of this manuscript.

**Response:** The Minority Stress Theory has been included as recommended by the reviewer to strengthen the contribution of this manuscript.

7. **Reviewer 2:** There are no explicit discussions on the impact of disclosure or nondisclosure on mental health of SMM.

**Response:** Although we did not explicitly ask questions targeting the impact of nondisclosure on the mental health of sexual minority men in our study, we have made appropriate inferences to studies that have directly or indirectly focused on non-disclosure-related mental health issues. These references establish a meaningful connection between our findings and the existing literature on mental health among sexual minority populations.

8. **Reviewer 2:**  The type of sampling used in this study is not explicitly specified, although it appears to be convenience sampling, with participants recruited from agencies working with MSM.

**Response:** We are pleased to note that you found the section on engagement with sexual minority men to be adequately discussed. Our research team employed a time-location sampling technique, a form of convenience sampling in collaboration with community partners who have established relationships with these individuals.

Additionally, we have reworded our manuscript title from "Family Rejection of Sexuality – Sexual and Gender Anonymity among Sexual Minority Men in Slum Communities" to Family rejection of non-hetero sexuality – sexual orientation and behavior anonymity among sexual minority men in slum communities-BSGH 001. The theme, "Disclosure fears of facing harm from family" has been reworded to "The fear of facing harm from family." The subtheme "Fear of deviation from community culture and expectation of gender roles and attitudes" has been modified to "Fear of deviation from community culture, gender norms, and expectations."

Furthermore, to ensure proper documentation, we have appended the manuscript number for our lab (BSGH 001) to the manuscript title, which was not previously included.

In conclusion, I want to express our sincere appreciation for your invaluable contributions to improving our paper. Your feedback has significantly enhanced the quality and of our paper. We have carefully addressed all the concerns raised by Reviewer One and Reviewer Two, and we believe that the revised version of the paper now exceeds the standards set forth for publication.

We genuinely hope that you find the revisions satisfactory and look forward to receiving your final evaluation and decision regarding the publication of our manuscript. Once again, we extend our deepest gratitude for your time, dedication, and valuable input throughout the review process.

Thank you,

**Osman Wumpini Shamrock PhD**

**Postdoctoral Research Associate**

**University of Rochester, School of Nursing**

**601 Elmwood Avenue, Box SON**

**Rochester, NY 14620**

**Phone: 585-276-5821**
