# Supplementary material for: Family rejection of non-hetero sexuality–Sexual orientation and behavior anonymity among sexual minority men in slum communities-BSGH 001
Source: PLOS Glob Public Health. 2023 Dec 1;3(12):e0001659. doi: 10.1371/journal.pgph.0001659 (PMC10691700; doi:10.1371/journal.pgph.0001659)
Supplement: Attachment — Submitted filename: Family and non disclosure of SMM.docx [file pgph.0001659.s003.docx]

Thank you for engaging me as a reviewer for "Family rejection of non-heterosexuality – sexual orientation and behavior anonymity among sexual minority men in slum communities”

I have read with pleasure this interesting paper. The paper reads very well, and it is a great contribution. The authors have adequately addressed my concerns.

A minor clarification though:

Page6: Research design is a qualitative phenomenological, but does not mention the techniques for data collection; however, on page 10 sub-title Fear of physical assault for family…- Line 5 it is mentioned- in depth interviews with SMM, kindly clarify in the research design whether in depth interview only data collection technique.
