# Supplementary material for: Family rejection of non-hetero sexuality–Sexual orientation and behavior anonymity among sexual minority men in slum communities-BSGH 001
Source: PLOS Glob Public Health. 2023 Dec 1;3(12):e0001659. doi: 10.1371/journal.pgph.0001659 (PMC10691700; doi:10.1371/journal.pgph.0001659)
Supplement: Attachment — Submitted filename: Reviewer Response .docx [file pgph.0001659.s004.docx]

Reviewer #1: A minor clarification though:
Page6: Research design is a qualitative phenomenological, but does not mention the techniques for data collection; however, on page 10 sub-title Fear of physical assault for family…- Line 5 it is mentioned- in depth interviews with SMM, kindly clarify in the research design whether in depth interview only data collection technique. –

Response: Thank you for the comment. We have clarified in the research design section that the in-depth interview data collection technique was employed approach was to understand the lived experience on disclosure , rejection, or acceptance of non-heterosexuality among SMM.

Reviewer #2: The authors have addressed most of the key comments and the revisions have strengthened the manuscript.

Family: In the Discussion section, the authors can discuss the possibility that SMM may be concerned that their family members might be harmed by others/society if they disclose their sexual orientation (an alternative/rival explanation or evidence for which may be available in their data). The findings only argue that SMM might be harmed by their family, so they do not disclose their sexual orientation to the family (or others). The authors can briefly speculate about the potential influence of religion on the family's attitudes, whether or in what ways the religion (or other factors) contributed to the proposed legislation, and the potential influence of the proposed legislation on the family’s attitudes toward SMM.

Response: We agree this is an strong point to consider when disclosure of sexual orientation/behavior are discussed. We have included this suggestion in the discussion section, and also in the implication sections where interventions are considered. We also acknowledge that significant role of religion on the influence on legislation in different context. However, in the case of our study area and country, there is no scientific connection to proof this phenomenon. We however, have included this strong point in the limitation and future research section of the paper.

Regarding the type of sampling: It appears to be convenience sampling as the authors recruited SMM from two slums and they did not use any time slots for recruitment. The authors need to explain why they consider it as a time-location sampling. If not, they need to revise the sentence on the type of sampling – retaining the term ‘convenience sampling’ but deleting the term “time-location sampling" or "TLS”. –

Response: We have given an expanded explanation to the TLS sampling techniques, and included in the text “The TLS technique allowed the research team to meet with SMM at specifics times and locations that were previously agreed with community organizations (Priorities on Rights and Sexual Health – PORSH) and Kumasi (Youth Alliance on Health and Human Rights – YAHR) in their monthly meeting schedules. The technique also allowed the research team to safely interview participants, and provide the sample with a diverse pool of respondents as SMM were randomly chosen from multiple visits at meeting times and locations not determined by the researchers.”
