# Supplementary material for: Family rejection of non-hetero sexuality–Sexual orientation and behavior anonymity among sexual minority men in slum communities-BSGH 001
Source: PLOS Glob Public Health. 2023 Dec 1;3(12):e0001659. doi: 10.1371/journal.pgph.0001659 (PMC10691700; doi:10.1371/journal.pgph.0001659)
Supplement: Attachment — Submitted filename: Response to Reviewer 2.doc [file pgph.0001659.s005.doc]

Dear reviewers,

We have addressed the following changes mentioned below with the following responses highlighted in yellow. The revised manuscript is titled “Revised manuscript 2”

1. It is preferred in social research not to speak of “proof” but rather something like evidence or support.

p. 22. Change this:

“While we acknowledge with proof that SMM avoided disclosing their sexual orientation/behavior to family due to the perceived risk of facing harm from their relatives. We also recognize that SMM may avoid disclosure in their communities to prevent attacks on their family members (Boulder, 2018; Lavietes, 2022).”

To something like this:

While we identified evidence that SMM avoided disclosing their sexual orientation/behavior to family due to the perceived risk of facing harm from their relatives, we also recognize that SMM may avoid disclosure in their communities to prevent attacks on their family members (Boulder, 2018; Lavietes, 2022). – We have accepted this suggestion and made the necessary changes as suggested by the reviewer. This have been changed to “While we identified evidence that SMM avoided disclosing their sexual orientation/behavior to family due to the perceived risk of facing harm from their relatives, we also recognize that SMM may avoid disclosure in their communities to prevent attacks on their family members (Boulder, 2018; Lavietes, 2022).”

2. Since you did not identify this in your own study, it is best to use more tentative wording (i.e., not “urge”); and the first phrase is unnecessary: Delete: “While these considerations are being considered….”Also, by protections do you mean resources to assist families? - We agree and realize this was a point raised by the second reviewer who suggest changes the sentence to “We also suggest strategies to consider and implement resources for families who are in support of their relatives’ sexual orientation/behavior.”

p. 27: Change this:

“While these considerations are being considered, we also urge for strategies to consider and place protections for families who are in support of their relatives’ sexual orientation/behavior.”

To something like this:

We also suggest strategies to consider and implement resources for families who are in support of their relatives’ sexual orientation/behavior. – We (author) agree with this comment and have changed the wording in this section to “We also suggest strategies to consider and implement resources for families who are in support of their relatives’ sexual orientation/behavior.”

Sincerely

Osman Wumpini Shamrock PhD

School of Nursing, University of Rochester

225 Crittenden Blvd, Rochester, NY, 14642

+1 (607) 761 8189
